# Natural Immunity to HIV: A Template for Vaccine Strategies

**DOI:** 10.3390/v10040215

**Published:** 2018-04-23

**Authors:** Lyvia Fourcade, Johanne Poudrier, Michel Roger

**Affiliations:** 1 Laboratoire d’Immunogénétique, Centre de Recherche du Centre Hospitalier de l’Université de Montréal (CRCHUM), Montréal, QC H2X 0A9, Canada; lyvia.fourcade@gmail.com; 2 Département de Microbiologie, Infectiologie et Immunologie de l’Université de Montréal, Montréal, QC H3C 3J7, Canada

**Keywords:** HIV, HESN, natural immunity, regulatory dendritic and T-cells, BLyS/BAFF, innate marginal zone B-cells

## Abstract

Africa accounts for the majority of global human immunodeficiency virus (HIV) infections, most of which affect women through heterosexual intercourse. Currently, there is no cure for HIV and the development of vaccines and microbicides remains the best solution to eradicate the pandemic. We and others have identified HIV highly-exposed seronegative (HESN) individuals among African female commercial sex workers (CSWs). Analyses of genital samples from HESNs have demonstrated potent innate and anti-inflammatory conditions, HIV-specific CD4^+^ and CD8^+^ T-cells as well as immunoglobulins (Igs), and increased regulatory cell populations, all of which support a delicate balance between strength and control against HIV intrusion. Moreover, we have recently shown that frequencies of innate marginal zone (MZ) B-cells are decreased in the blood of HESNs when compared to HIV-uninfected non-CSW women, suggesting their recruitment to peripheral sites. This coincides with the fact that levels of B lymphocyte stimulator (BLyS/BAFF), known to shape the MZ pool and whose overexpression leads to MZ deregulation in HIV-infected progressors, are significantly lower in the blood of HESNs when compared to both HIV-infected CSWs and HIV-uninfected non-CSW women. Interestingly, MZ B-cells can bind HIV gp120 and produce specific IgG and IgA, and have a propensity for B regulatory potential, which could help both the fight against HIV and maintenance of low inflammatory conditions in HESNs. HESN individuals provide an exceptional opportunity to identify important clues for the development of protective devices, and efforts should aim at soliciting immune responses observed in the context of their natural immunity to HIV.

## 1. Introduction

Worldwide, it is estimated that nearly 36.7 million people live with human immunodeficiency virus (HIV). In 2016, around 1.8 million became newly infected and 1 million died from AIDS. Africa accounts for 69% of global infections, most of which affect women through heterosexual intercourse [1]. Currently, there is no cure for HIV and the development of preventive strategies such as vaccines and microbicides remains the best solution to eradicate the pandemic. To date, the transmission mechanisms of the virus and immune responsiveness at the initial site of infection are not fully understood. Frequent mucosal exposure to HIV in the absence of infection was documented in different cohorts, including the Beninese commercial sex workers (CSWs) [2]. As such, individuals highly exposed to HIV and persistently seronegative (HESN) have been shown to possess low-inflammatory conditions and immune responsiveness towards the virus [2,3,4], which suggests that the capacity to maintain a low-key inflammatory profile along with anti-HIV responses is associated with protection against HIV infection. We believe that efforts to develop effective devices should aim at mimicking conditions and soliciting immune responses observed in the context of natural immunity to HIV.

## 2. Immunology of the Female Genital Tract

The female genital tract (FGT) is part of the major mucosal associated lymphoid tissue (MALT) [5]. The FGT constitutes a main portal of entry for many organisms and plays a role in protecting the host against pathogens while maintaining a tolerance to a commensal flora [5,6]. FGT immunity is tightly regulated by a hormonal/inflammatory process throughout the menstrual cycle, having to deal with the pressure of procreation and microbial control [7,8]. FGT is subdivided into 2 regions presenting distinct phenotypic profiles. The upper FGT consists of the sterile endometrium, fallopian tubes and the endocervix in which sterility may be temporally related to the menstrual cycle phase. In contrast, the lower FGT, which is composed of the non-sterile vagina and ectocervix is colonized by a commensal microflora [8]. FGT immunity involves genital epithelial cells (ECs) as well as dendritic cells (DCs), Langerhans cells (LC), macrophages, natural killer (NK) cells, neutrophils and lymphoid cells, which confer protection through the production of antimicrobial agents, antibodies, chemokines and cytokines [5]. Wira and colleagues have shown that the upper FGT contains unique lymphoid aggregates constituted of CD8^+^ T cells that surround a central B-cell core, which are encapsulated by macrophages [7]. Even if mechanisms of immune induction in the FGT remain poorly understood [2,6,7], the FGT is provided with an array of protective mechanisms from the innate and adaptive arms of the immune system to maintain a delicate balance between protection and tolerance [9].

Together with ECs, DCs are one of the earliest cell types to sense the virus through pattern recognition receptors (PRRs), such as toll-like receptors (TLRs), lectins and NOD-like receptors [2,10,11]. Cross-talks between ECs and sub-mucosal DCs involve immunomodulatory cytokines and lead to activation of effector and regulatory cells in the lamina propria [2,11]. It is well known that DCs are important for the generation of first-line innate as well as adaptive immune responses [11] during infections. Indeed, DCs are involved in the delivery of cognate and non-cognate molecular events as well as production of immunomodulatory molecules, such as cytokines and growth factors that can shape the overall outcome of T and B lymphocyte responses [11].

## 3. The Female Genital Tract in the Context of HIV

The study of the FGT in CSWs is often complicated by numerous difficulties associated with ethics and sampling, and therefore studies are often guided by observations from the gastro-intestinal lymphoid tissue (GALT) [12]. The FGT involves the mucous lining of a tight EC barrier, stratified at the vaginal and ectocervical levels [7]. Integrity of ECs can be influenced by pro-inflammatory factors such as tumor necrosis factor (TNF) [13] but also by sustained Th17 cell activity [14] in the context of early HIV infection. This could affect tight junctions of mucosal ECs leading to increase risks of microbial translocation and chronic infection [12], and also facilitate virus transcytosis across ECs [13,15,16], particularly via the galactosylceramide (GalCer) receptor [17]. In fact, although HIV does not productively infect ECs [2], GalCer, a glycosphingolipid highly enriched at the luminal pole of ECs, can bind both HIV glycoproteins gp120 and gp41 [18], and allows endocytosis by ECs, which subsequently transfer the virus to DCs and target cells [2,19,20]. HIV has also been shown to be internalized by FGT ECs via gp340, a scavenger receptor, subsequently promoting the production of pro-inflammatory thymic stromal lymphopoietin (TSLP) via TLR7 signaling, which then activates DCs and promotes HIV transmission to CD4^+^ T cells [21].

Although DCs can be infected by HIV, mostly at an immature stage, they express potent restriction factors such as SAMHD1 [22] and do not constitute the main target for the virus and are rather involved in its transmission to CD4^+^ T-cells [23]. Sub-mucosal DCs express high levels of C type lectins, such as DC-SIGN (CD209), which can bind gp120 [20,24]. This allows internalization of the virus and transfer to CD4^+^ CCR5^+^ effector target T-cells, either locally or following migration to draining lymphoid organs [25].

Using simian immunodeficiency virus (SIV)/macaque vaginal infection model [26,27], numerous infectious foci throughout the FTG were identified early after infection. It has been shown that CCR6^+^ Th17 cells are the preferential targets of SIV following vaginal transmission [26,27]. These Th17 targets express RORγt, a transcriptional regulator required for their generation and differentiation [28,29]. Th17 cells also express α4β7 another co-receptor for HIV [30]. Factors such as transforming growth factor (TGF)-β, interleukin (IL)-1, IL-6, IL-21 and IL-23 are required for Th17 differentiation [28]. CCR6 is a major ligand for the chemokine macrophage inflammatory protein-3alpha (MIP-3α/CCL20), which is secreted by mucosal ECs and is known to attract immature LCs and DCs [31,32]. It has been shown that strong doses of SIV in the vaginal mucosa caused an increase of the chemokine MIP-3α/CCL20 [19] and recruitment of plasmacytoid DCs, DCs and macrophages at the cervical epithelium [23]. Early blocking of MIP-3α and pro-inflammatory cytokines prevented cellular recruitment, establishment of an inflammatory milieu, and infection despite repeated intravaginal exposure to high doses of SIV [16,33]. CD4^+^ T cells can also be recruited via MIP-1α/CCL3 and MIP-1β/CCL4. Interferon (IFN)-α is an important promotor of CD4^+^T cells clonal expansion at the vaginal mucosa, and subsequently in the blood and secondary lymphoid organs [19]. Establishment of systematic infection eventually leads to a massive depletion of sub-mucosa CD4^+^ T cells, in particular Th17 cells (CCR4^+^CCR6^+^), that is associated with an increased regulatory T (Treg) cells, leading to an imbalance in the ratio of T effector vs Treg cell populations [2,14,16,23].

## 4. Natural Immunity to HIV in the FGT of HESNs

Blood and genital mucosal factors that constitute a low-inflammatory “immune” profile have also been linked with protection in HESNs [2,34,35]. Indeed, it has been shown that high levels of anti-inflammatory and neutralizing proteins, such as anti-proteases are found in the genital mucosa of HESN CSWs [2,4,34]. HIV-Env reactive immunoglobulins (Igs) have also been documented in blood and FGT of HESNs, and will be discussed later in this review. Levels of pro-inflammatory cytokines such as IL-1α, IFN-γ and TNF-α, as well as monokine induced by IFN-γ (MIG) and IFN-γ induced protein (IP)-10 chemokines have been reported to be lower in cervicovaginal lavages (CVLs) of HESNs when compared to HIV-infected CSWs [36,37]. In fact, production of MIG and IP-10 is induced by expression of IFN-γ, and polymorphisms in the IRF-1 regulating IFN-γ were associated with protection to HIV [38,39]. In a Kenyan female CSW cohort, HIV-specific CD4^+^ and CD8^+^ T-cell responses have been found in both the blood and genital tract of HESN CSWs [40,41,42,43]. In these individuals a low activation T-cell profile corresponds with a greater ability to proliferate in response to HIV p24 peptides when compared to that observed in HIV-infected CSWs [44]. Moreover, these studies also identified poly-functional effector T-cells in HESNs.

Interestingly, we have shown that Beninese HESN CSWs presented higher levels of IFN-α in their CVLs when compared to those observed in HIV-infected CSWs and HIV-uninfected non-CSWs [45], which could be critical to sustain immune homeostasis, antiviral activity and restriction factors such as SAMHD1, APOBEC3, or tetherin in cells at the portal of entry for the virus. Indeed, type I interferons are indispensable to protect host against viruses [46]. Although in uncontrolled situations such as in the context of HIV-infection, type I IFNs promote inflammatory responses as well as recruitment of target cell, they can also induce a myriad of IFN-stimulated genes (ISGs), which have been shown to interfere with multiple viruses at various life cycle stages [47]. Type I IFNs can be triggered via TLRs 3, 7, 8 and 9 [48], and HIV ssRNA triggers TLR 7 and TLR 8 [15,46]. Interestingly, genital epithelial and myeloid cell populations of Beninese HESN CSWs expressed high levels of TLR 7 [45]. IL-10, which is known to promote immunoregulatory responses was elevated in the CVLs of Beninese HESNs, but lower than that observed in CVLs of HIV-infected CSWs [45]. IL-10 levels are often elevated in the context of HIV, unfortunately the overall outcome of excessive IL-10 may well be to sustain chronic activation and dysregulation associated with HIV disease progression, and may impede on viral eradication [49,50,51]. A more modest elevation of IL-10, such as observed for HESN CSWs, may be beneficial and promote an immunoregulatory microenvironment preventing HIV attempts to establish infection by lowering availability of targets [52]. Therefore, IFN-α and IL-10 levels measured in the CVLs of Beninese HESN CSWs likely promote a potent antiviral and yet at the same time immunoregulatory conditions.

## 5. Immunoregulatory Cell Populations in HESNs

Studies of the genital immune profile in HESN CSWs suggest that natural immunity in the context of HIV may be associated with the host’s capacity to orchestrate dynamics of cellular populations to maintain low inflammatory conditions at the initial site of exposition. In this view, we recently reported that endocervical myeloid HLA-DR^+^ cells from Beninese HESN CSWs expressed higher levels of IFN-α, TLR 7, IL-10 and HLA-G than those from both HIV-infected CSWs and HIV-uninfected non-CSWs. Further characterization of these cells in HESNs revealed a CD103^+^ CD14^+^ CD11c^+^ “DC-like” population expressing high levels of IFN-α and IL-10 [45], which is reminiscent of the recently described tolerogenic profile of “DC-10” [53]. Interestingly, the majority of the myeloid CD11c^+^CD14^+^IFN-α^+^IL-10^+^ cells also expressed HLA-G and ILT-4 [45], as do DC-10 [53,54]. Concomitantly, Beninese HESN CSWs had higher frequencies of endocervical regulatory CD4^+^ T-cells (Tregs) when compared to those from the two other groups of women [45], which is consistent with that reported in the blood of Kenyan CSWs [55]. Moreover, we found an increased frequency of endocervical Tregs expressing programmed cell death protein (PD)-1^+^, as well as higher intensity of PD-1, IL-10 and CTLA expression for both Tregs and type 1 regulatory cells (Tr1) (CD49b^+^ and LAG-3^+^) [56] in Beninese HESNs [45]. This could be reflective of their ongoing regulatory activity [57], which likely confers an advantage to these individuals.

Thus, to date, observations on HESNs suggest that natural immunity against HIV involves a capacity to induce/maintain strong innate and HIV-specific immune responses, and at the same time, regulatory populations such as “DC-10-like”, Treg and Tr1 to help control/maintain low inflammatory conditions at the initial site of exposure. Understanding how the potent antiviral but regulated inflammatory response observed in HESNs is achieved at the initial site of infection is crucial for the development of effective mucosal microbicides or vaccines.

## 6. The Importance of HIV ENV Reactive Immunoglobulins: Lessons from Vaccination Trials

Although the induction of broadly neutralizing antibodies (bNAbs) is a main goal in vaccination [58], there is growing evidence suggesting that both neutralizing and non-neutralizing Abs can mediate some level of protection against HIV [59]. Analyses of correlates of protection from the RV144 vaccine trial suggested that increased blood derived IgG1 and IgG3-mediated Ab dependent cell cytotoxicity (ADCC) activity towards HIV ENV V1V2 region was linked with decreased HIV acquisition [60]. Genetic analyses demonstrated that RV144 vaccinees bearing HLA class II alleles such as DQB1*06 presented increased risk of HIV acquisition possibly associated with elevated ENV-specific IgA interfering with ADCC activity [59,60]. However, non-neutralizing ENV-reactive IgA derived from blood memory B-cells of RV144 vaccinees who did not bear predisposing HLA alleles, blocked in vitro HIV ENV binding to GalCer and mediated in vitro phagocytosis by monocytes expressing FcRα [59]. Raising the possibility that the RV144 regimen may have induced a certain level of non-neutralizing, protective IgA in some individuals [59]. Albeit, one of these ENV reactive IgA antibody did not bind to the RV144 immunogen, and was possibly derived from a pre-existing B-cell pool, expanded by vaccination and microbiota reactive [59], as it has been shown that some HIV ENV reactive Abs cross-react with microbiota [61].

Samples from mucosal ports of entry were not collected during the RV144 trial and the reactivity of mucosal Igs have not been assessed. The importance of mucosal Igs, in particular IgA, in fighting HIV has been highlighted by vaccination and passive immunization studies [62]. At mucosal sites, IgA is produced in the lamina propria by local plasma cells, mainly as dimeric (dIgA) containing a joining J chain. It can translocate across ECs to generate luminal secretory IgA (SIgA) via the polymeric Ig receptor (pIgR). Mucosal IgA can mediate protection by trapping, neutralizing and preventing transcytosis [62]. In 2011, the elegant study by the group of Morgan Bomsel had shown that vaccination of non-human primates with HIV gp41 virosomes induced mucosal IgA and IgG, which prevented systemic invasion following vaginal simian-HIV challenge, by blocking transcytosis and by mediating ADCC, respectively [63]. The fact that these animals lacked serum neutralizing antibody activity highlighted the importance of effector antibodies at the mucosal portal of entry [63]. More recently, passive immunization studies showed that rhesus macaques given anti-HIV-1 neutralizing monoclonal Ab (NmAb) HGN194 as mucosal dIgA2 together with systemic IgG1 with the same epitope specificity were completely protected against high-dose intra rectal SHIV-1157ipEL-p challenge [64,65]. Furthermore, the dIgA1 version of the same mAb was significantly more protective compared to the dIgA2 version, highlighting the importance of characterizing different isotypes of IgA, as they differ predominantly in the hinge region and may confer varying effector functions [62]. The fact that these NmAb were poorly efficient when used alone implies that inducing mucosal IgA as first-line defense in conjunction with immunity against HIV at the systemic level is required to prevent virus acquisition [64,65].

## 7. HIV ENV Reactive Immunoglobulins in HESNs

Given the 31% HIV protection conferred by the RV144 vaccine regimen, we were inclined to screen sera and genital samples from the Benin CSW cohort for the presence of anti-HIV trimeric ENV IgG as well as for their potential to neutralize HIV viral particles and/or mediate ADCC. Although anti-HIV ENV IgG, neutralizing or ADCC activities were detected in samples from HIV-infected CSWs, no such activities were observed in blood and CVLs from HESN CSWs [66]. Therefore, suggesting that natural immunity may not be associated with the production of HIV-specific IgG mediating neutralizing or ADCC activities. We and others have detected ENV reactive IgA in HESN individuals, which may help prevent HIV infection [37,62]. Indeed, it has been shown that HESNs present HIV cross-clade neutralizing-IgA in their blood and FGT, which are mostly directed towards ENV glycoproteins [67,68,69,70,71]. Furthermore, in a cohort of HESN women from Ivory Coast, mucosal IgA were shown to block HIV transcytosis through tight epithelial barriers [72,73]. Whether these IgA are generated and maintained in response to frequent HIV exposition and/or a contained local HIV reservoir and/or expanded from pre-existing microbiota reactive B-cell pools that cross-react with HIV-ENV remains to be established. The sol fact that sex-break will eventually lead to seroconversion in HESN CSWs who return to sex work, despite pre-existing HIV-specific responses [74], suggests frequent exposition to HIV is required for maintenance of immune populations and their protective responses in the mucosal niche. This could possibly operate via a mechanism analogous to that described in the GALT in the context of host-microbiota immune “tolerance” system [75].

## 8. Innate B-Cells and BLyS/BAFF in Natural Immunity to HIV

Until now, few studies have assessed the nature of B-cells involved in production of Abs in the context of natural immunity against HIV. The detailed characterization of the Ig repertoire of cervical and systemic B-cells from a Kenyan HESN individual revealed that site-specific responses occur with unique regulation of tolerance and recruitment into local memory or blast B-cell compartments, and the infusion of systemic post-germinal center (GC) B-cells to the cervix seems to be a common event [76]. Further understanding the nature and how B-cell populations are solicited to fight against HIV appears important to the design of preventive approaches.

Growing importance is given to innate marginal zone (MZ) type B-cells in health and disease [77]. Indeed, given their location in lymphoid organs and mucosal-associated structures, human MZ B-cells constitute early first-line defense against invading pathogens. Although the Abs generated from innate B-cells may be important in some circumstances, such as with MZ B-cells in the context of encapsulated bacteria [77], in others such as with viral infections more refined adaptive Ab responses are also required to eradicate the infection. In viral infections, innate populations such as MZ B-cells likely provide a quick efficient first-line response and participate in the development of the adaptive Ab responses. Indeed, MZ B-cells can traffic Ag to follicular B-cell areas of lymphoid structures and promote GC reactions, where T-cell dependent B-cell class switch recombination (CSR) and affinity maturation lead to the production of highly specific Abs with potent neutralizing and ADCC effector functions [78]. MZ B-cells are capable of CSR, mostly towards IgG and IgA in humans [77]. MZ B-cells express a polyreactive BCR repertoire, which comprises usage of the IGHV1-2 gene [79], whose product has been shown to take part to HIV-ENV reactive broadly neutralizing Abs (bNAbs) such as VRC01 [80]. Moreover, human MZ B-cells have been shown to naturally bind to fully glycosylated gp120 via surface lectins, and in presence of BLyS/BAFF subsequently produced polyreactive IgG and IgA, of which a fraction recognized gp120 [81].

BLyS/BAFF is highly recognized for its role in B-cell ontogenesis, as well as cell fate decision towards the innate MZ B-cell pool [77,82]. Interestingly, repeated treatment of mice with BLyS/BAFF increased their MZ B-cell compartment and generated an increased response to ENV immunization and bNAbs [83]. Although the capacity of MZ B-cells to bind gp120 suggest they could possibly transfer HIV, it is unlikely that they get infected by the virus. Indeed, despite the fact that HIV has been shown to replicate in CD40 stimulated B-cells in vitro [84], the virus has not yet been convincingly shown to infect or replicate in B-cells in vivo [85]. Moreover, HiSeq gene expression analysis of MZ B-cells reveals relatively high levels of viral restriction factors such as SAMHD1 (Poudrier J and Roger M, preliminary data 2016).

Previous reports by us and others demonstrated that BLyS/BAFF expression is increased in the context of HIV disease, and not fully restored following therapy [86,87]. This is likely due to direct and indirect factors associated with HIV infection. Indeed, soluble HIV-Nef can directly modulate BLyS/BAFF membrane expression and soluble release by monocyte derived DCs [88], and HIV-ENV has been shown to upregulate BLyS/BAFF expression by macrophages [81]. Furthermore, BLyS/BAFF has been shown to be directly induced by type I IFNs [89,90]. Elements of microbial translocation, such as LPS are also known to up-regulate BLyS/BAFF expression and release [88,91]. We have previously shown that BLyS/BAFF overexpression in blood of HIV-infected progressors coincides with hyperglobulinemia and increased frequencies of IL-10 expressing precursor-like MZ B-cells [50,86], which HiSeq gene expression analyses reveal several dysregulated genes (Poudrier J and Roger, preliminary data 2016). We also found overexpression of BLyS/BAFF and increased frequencies of precursor-like MZ B-cells in the blood of HIV-infected CSWs from Benin [92]. Elevated BLyS/BAFF levels likely favor expansion, activation and dysregulation of innate B-cell populations such as MZ, contributing to the over-representation of polyreactive and auto-reactive Abs [93] at the expense of eradicative anti-HIV Ab responses. Interestingly, cumulating evidence point to the fact that increased BLyS/BAFF and dysregulated B-cells sharing similar features with the precursor-like MZ B-cells we identified in the blood of HIV-infected individuals are a common trait of chronic inflammatory diseases [94].

In contrast to that observed in HIV-infected progressors, both blood BLyS/BAFF levels and frequencies of precursor-like MZ B-cells remained unaltered in HIV-infected Elite-Controllers [50,86]. Rather, frequencies of more mature MZ B-cells were found to be decreased in blood, implying that their recruitment to periphery, as suggested by their elevated migratory potential [95], may be involved in control of HIV disease progression. Although the mechanisms conferring natural immunity against HIV remain to be elucidated, we hypothesize they may share some similarities with that we observed in HIV-infected Elite-Controllers. Accordingly, in the Benin cohort, plasma and cellular BLyS/BAFF levels were significantly lower in the blood of HESN CSWs when compared to those measured in HIV-infected CSWs and HIV-uninfected non-CSW women [92]. These low BLyS/BAFF levels are consistent with the low-inflammatory response we have previously described in these individuals [37], and may be linked to the modulation of the intracellular machinery leading to BLyS/BAFF expression and/or release. Frequencies of precursor-like MZ B-cells remained unaltered in HESNs when compared to HIV-infected CSWs [92]. However, as for HIV-infected Elite-Controllers, frequencies of more mature MZ B-cells were decreased in their blood when compared to HIV-uninfected non-CSWs [92]. It is conceivable that these cells are recruited to the periphery, where they generate first-line responses against HIV. Moreover, given their propensity for B regulatory potential [50] they could also contribute to the maintenance of low inflammatory conditions observed in HESNs. Furthermore, since MZ B cells promote GC reactions, it is likely that they also participate in the development of adaptive B-cell responses against HIV. Therefore, immunomodulation of BLyS/BAFF levels and innate MZ-like B-cell populations may benefit to mucosal preventive devices viewed to produce a rapid and potent first line antiviral immune response at the initial site of exposure, and which could be combined with refined adaptive immunity. Understanding the dynamics of BLyS/BAFF and its role in homeostasis of immune responsiveness appears pivotal to the design of vaccine strategies soliciting first-line B-cell responses. Based on our observations, the capacity to contain BLyS/BAFF expression levels seems concomitant with natural immunity against HIV, whereas excessive BLyS/BAFF may promote immune dysregulation and disease progression.

## 9. Concluding Remarks

**Figure 1:**
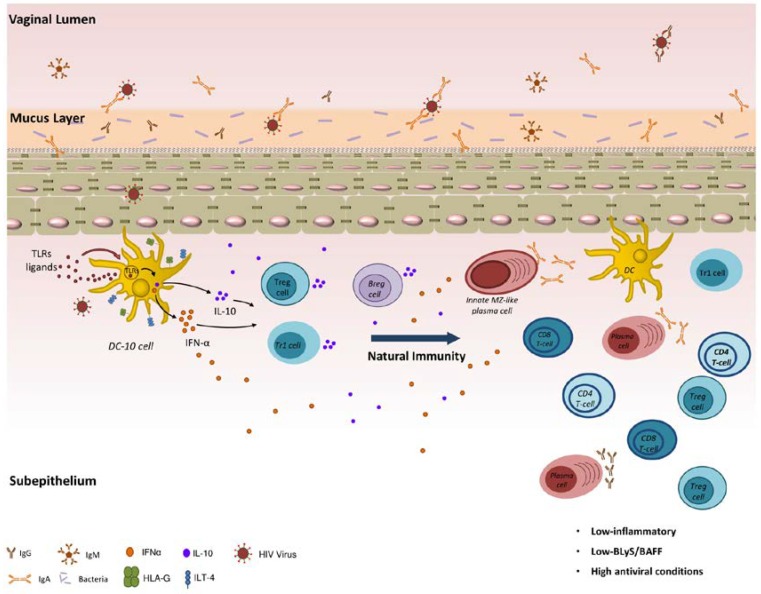
Natural genital mucosal immunity to HIV.

As depicted in Figure 1, natural genital mucosal immunity to HIV in HESN CSWs likely implies a strong capacity to generate efficient anti-viral responses and at the same time to prevent excessive inflammation. It likely involves orchestration of first-line innate immune responses in conjunction with matured high-affinity adaptive responses, which is expected to operate at cervicovaginal mucosal sites, which are ports of entry and replication for the virus. Promotion of regulatory DC-10-like, Treg, Tr1 and possibly Breg cells locally may contribute to the maintenance of a low-inflammatory genital milieu. This allows balanced responses from effector populations. The fact that BLyS/BAFF levels are contained helps to maintain the integrity of innate, possibly MZ-like, B-cell responses, the latter of which likely produce IgG and/or IgA capable of binding to HIV Env.

Understanding the nature and how immune populations are recruited and maintained within mucosal niche to fight HIV is important to the design of effective preventive/therapeutic approaches. This is critical, especially for innate first-line B-cell populations, such as MZ, which do not generate memory, and possibly require a frequent degree of Ag exposure to be maintained in the mucosal niche. This suggests that any protective device soliciting first-line responses will likely require frequent boosting to the vaccine regimen.
